# The Effect of the Revision of the Carcass Trading Standard of Pigs on the Profitability and Growth Performances on Japanese Commercial Farms

**DOI:** 10.1111/asj.70150

**Published:** 2026-01-20

**Authors:** Tsubasa Suzuki, Katsumasa Kure, Mitsugi Ito, Yosuke Sasaki

**Affiliations:** ^1^ Department of Agriculture, School of Agriculture Meiji University Kawasaki Kanagawa Japan; ^2^ Value Farm Consulting Ibaraki Japan; ^3^ Akabane Animal Clinic, Co. Ltd Tahara Aichi Japan

**Keywords:** carcass weight, feed costs, margin over feed cost, sales revenue

## Abstract

This study investigated the effect of the revision of Japan's Carcass Trading Standard for Pork (CTSP) in January 2023, which increased the optimal carcass weight (CWT) range by 3 kg, on the profitability and growth productivity of commercial pig farms. We analyzed data from 116 Japanese farrow‐to‐finish farms in 2022 and 2023. The study found that the mean CWT significantly increased from 76.1 to 77.1 kg (*p* < 0.05), with 77% of farms increasing their CWT. Statistical analysis revealed that farms that had increased their CWT by more than 2 kg saw a significantly higher increase in margin over feed cost per market pig compared with farms that had decreased their CWT (*p* < 0.05). This increased profit was primarily driven by higher sales revenue per market pig owing to the increased CWT, while feed cost per pig was statistically similar across all change groups. In conclusion, the CTSP revision successfully encouraged most farms to increase CWT, providing a clear economic advantage in terms of margin over feed cost.

## 1 Introduction

Shipping pigs at their optimal weight is crucial for improving profitability (Van den Broke et al. 2019). In Japan, the key guideline for determining shipping weight is the carcass trading standard for pork (CTSP) established by the Japan Meat Grading Association (JMGA). CTSP determines carcass grade based on carcass weight and backfat thickness (JMGA 2018) and is designed to objectively evaluate carcass quality and ensure fair trade between farmers and wholesalers. As the carcass price differs for each grade at markets (Tokyo Meat Market Co. Ltd. 2025), it is important to manage the carcass weight and backfat thickness. On January 1, 2023, the CTSP was revised, with the carcass weight range for all grades increasing by 3 kg (JMGA 2022), in response to the Livestock Improvement and Breeding Targets (Ministry of Agriculture, Forestry and Fisheries of Japan 2020) to raise the carcass weight per market pig (CWT). The CTSP revision was also prompted by the increase in CWT over 26 years since the last change of CTSP. From 2003 to 2022, the average CWT gradually increased from 76.8 to 78.0 kg (Ministry of Agriculture, Forestry and Fisheries of Japan 2024). This increase may be attributed to genetic improvement and advances in management (Suzuki 2020).

The CTSP revision was considered to have induced changes in shipping strategies, such as an increase in CWT. However, an increase in CWT leads to an increased age at slaughter and cost per market pig (Davoudkhani et al. 2020; Malgwi et al. 2022). However, its effect on profitability and growth productivity has not yet been quantified. Therefore, this study aims to investigate the effect of the CTSP revision on profitability and growth productivity on Japanese commercial farms, by comparing these measurements in 2022, before the CTSP revision, with those in 2023, after the CTSP revision.

## 2 Materials and Methods

### 2.1 Data

For this study, annual data were obtained from the 123 Japanese farrow‐to‐finish commercial farms in 2022 and 2023 that had participated in the benchmarking program conducted by the Japanese Association of Swine Veterinarians (JASV). The data used in this study were the total number of market pigs, total CWT, total sales revenue, total feed cost, total amount of feed purchased, and the number of pigs reared and dead. In this study, data on market pigs combined both female and castrated pigs. The profitability measurements were expressed in Japanese yen (JPY), with an assumed exchange rate to the US dollar and euro of 150 and 175 JPY, respectively, in September 2025.

Based on these data, the measurements of profitability and growth productivity in 2022 and 2023 were calculated as follows:

1. CWT (kg) = total CWT/total number of market pigs,
2. Sales revenue per market pig (JPY) = total sales revenue/total number of market pigs,
3. Carcass price (JPY/kg) = total sales revenue/total CWT,
4. Feed cost per market pig (JPY) = total feed cost/total number of market pigs,
5. Feed price (JPY/kg) = total feed costs/total amount of feed purchased,
6. Margin over feed cost per market pig (JPY) = sale revenue per market pig‐feed cost per market pig (JPY)
7. Postweaning mortality (%) = total number of pigs dying after weaning/total number of pigs weaned,
8. Carcass‐based FCR = total amount of feed purchased/total CWT,
9. Age at slaughter (days) = 365 days/fattening efficiency (calculated as total number of market pigs/total number of pigs reared).

Growth productivity indicators in this study include age at slaughter, postweaning mortality, and carcass‐based FCR. All data were checked for missing or invalid records, with seven farms being removed from this study because of invalid records, leaving the data from 116 farms for use in this study.

A questionnaire survey was also conducted to determine any changes in the shipping strategy after the CTSP revision. The survey was conducted anonymously, so that there was no link to individual data for profitability and growth productivity from the 116 farms.

### 2.2 Statistical Analysis

The paired *t*‐test was used to compare the measurements of profitability and growth productivity between 2022 and 2023. A linear mixed‐effects model using the MIXED procedure with Tukey–Kramer multiple comparison tests was also used to investigate the effect of the changes in CWT between 2022 and 2023 (CWT‐Change). To minimize the effect of differences in economic indicators between 2022 and 2023, the response variables used in the model were the difference in the measurements of profitability and growth productivity between 2022 and 2023. This difference‐based approach was used to isolate the effect of CTSP revision, although macroeconomic factors (e.g., inflation) might still have residual influence. The explanatory variables were CWT‐Change, which classified the farms into those with a decrease in CWT in 2023 (*N* = 34), those with an increase in CWT of 0–1 kg in 2023 (*N* = 28), those with an increase in CWT of 1–2 kg in 2023 (*N* = 27), and those with an increase in CWT of more than 2 kg in 2023 (*N* = 27), and the CWT in 2022, which classified the farms based on the upper and lower 25th percentiles: less than 74.3 kg (*N* = 29), 74.3–77.6 kg (*N* = 58), and over 77.6 kg (*N* = 29). The average sow inventory was included as a covariate, and farm region (Hokkaido and Tohoku (*N* = 36), Kanto (*N* = 39), Kyusyu (*N* = 19), and others (*N* = 22)) was included as a random effect, with *p*‐values of less than 0.05 being considered significant. SAS software Version 9.4 (SAS Institute Inc., Cary, NC, USA) was used for all statistical analyses.

## 3 Results

**FIGURE 1:**
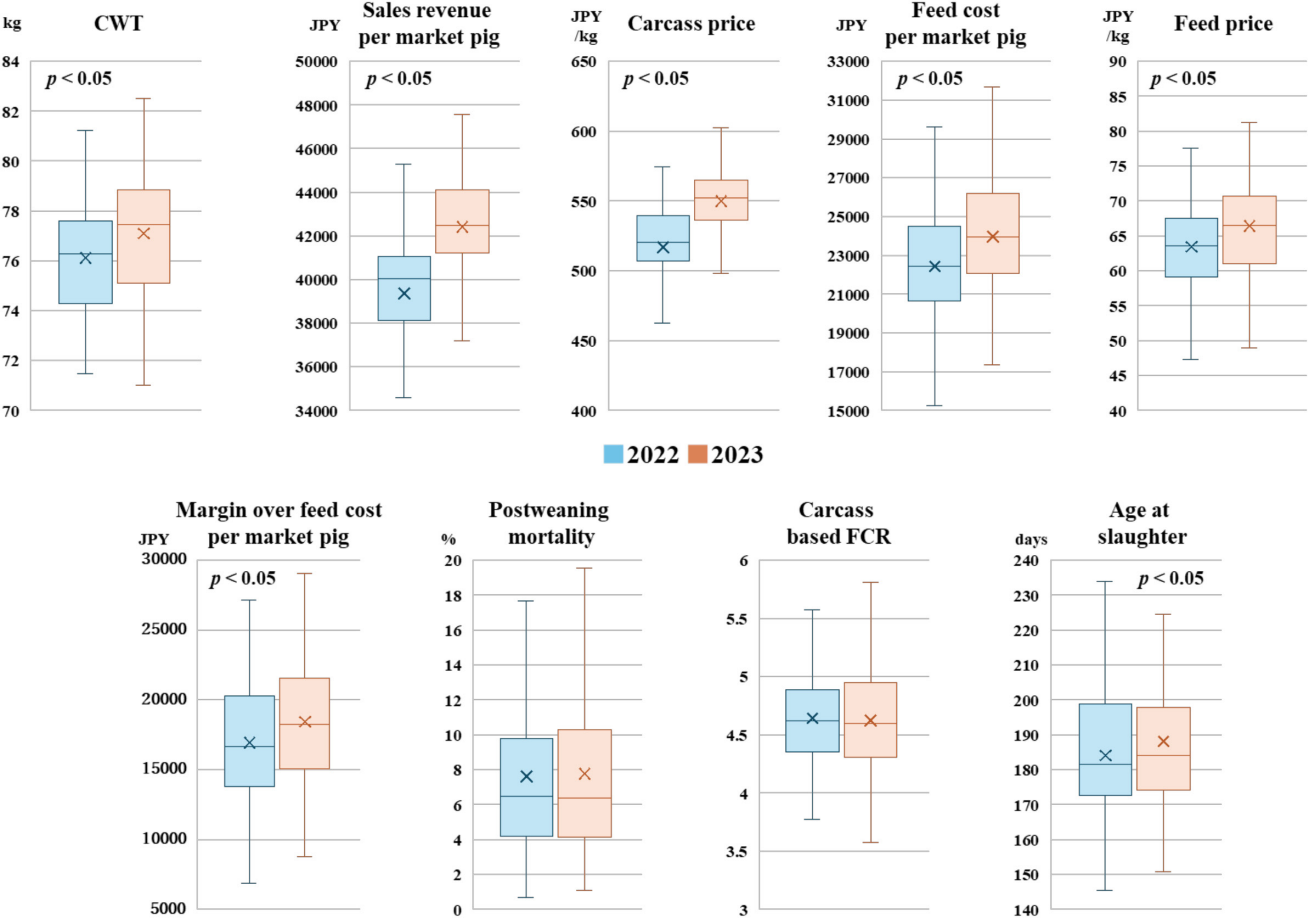
Comparisons of measurements of profitability and growth productivity between 2022 and 2023. *p* < 0.05 indicates a significant difference between years. CWT indicates carcass weight per market pig. Profitability measurements were expressed in Japanese yen (JPY).

Figure 1 shows comparisons of measurements of profitability and growth productivity between 2022 and 2023. The mean (±SD) CWT in 2023 was higher than that in 2022 (77.1 ± 0.2 kg vs. 76.1 ± 0.2 kg; *p* < 0.05). Of the 116 farms, 77% increased their CWT between 2022 and 2023; 8% by over 3 kg, 16% by 2–3 kg, 24% by 1–2 kg and 29% by 0–1 kg, whereas of the 23% with a decreased CWT, 9% exhibited a decrease of less than 1 kg and 14% a decrease of 0–1 kg. Between 2022 and 2023 the sales revenue per market pig increased significantly (*p* < 0.05). The age at slaughter increased between 2022 and 2023, and the feed cost per market pig increased in 2023 (*p* < 0.05). However, the margin over feed cost per market pig increased in 2023 compared with that in 2022 (*p* < 0.05). No significant differences were found between 2022 and 2023 in postweaning mortality and in the carcass‐based FCR.

**FIGURE 2:**
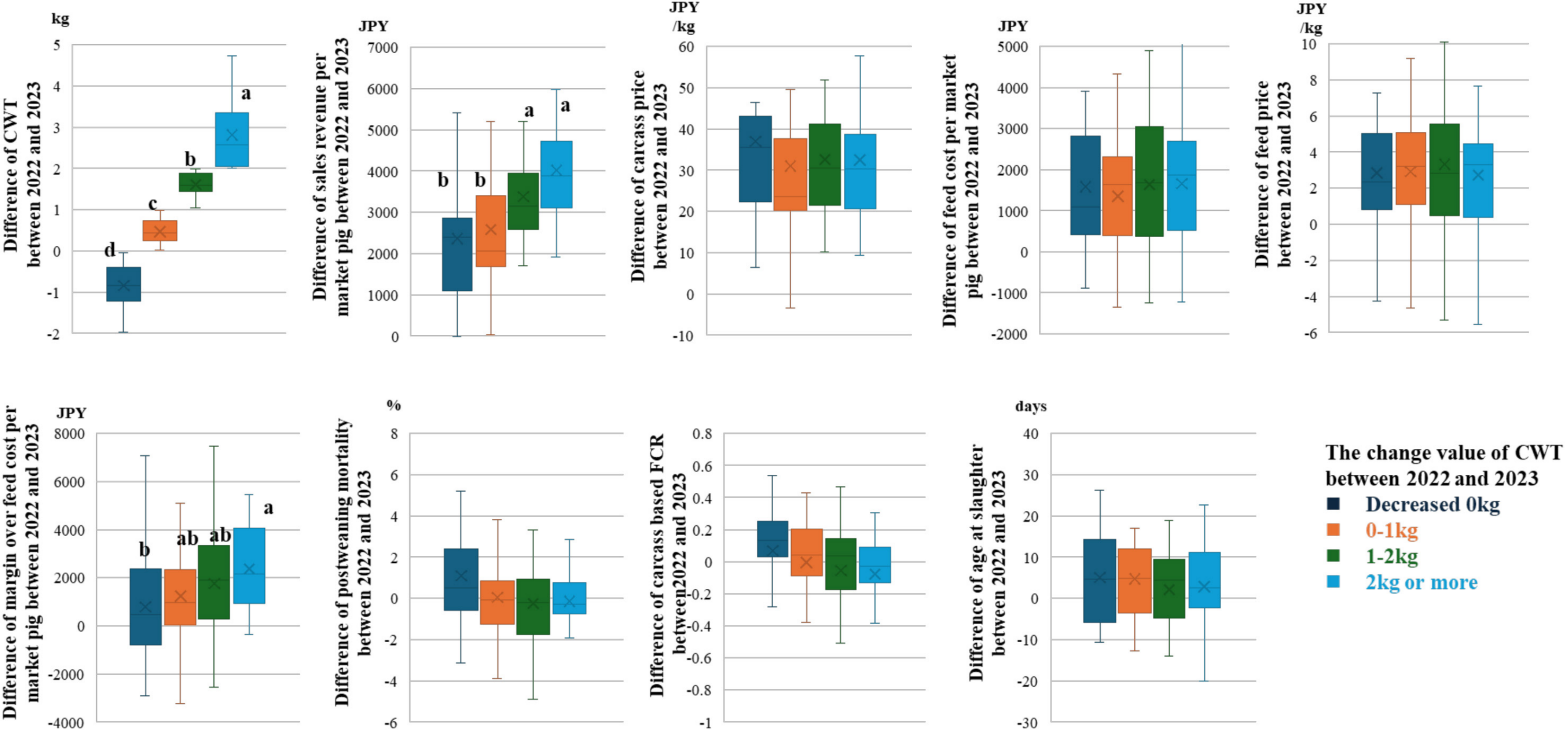
Comparison of the difference in values between 2022 and 2023 of the measurements of profitability and growth productivity among the change value of carcass weight per market pig (CWT) between 2022 and 2023 (CWT‐Change). The CWT‐Change was classified as farms that decreased CWT in 2023 (*N* = 34), farms that increased CWT by 0–1 kg in 2023 (*N* = 28), farms that increased CWT by 1–2 kg in 2023 (*N* = 27), and farms that increased CWT by over 2 kg in 2023 (*N* = 27). Values with different letters indicate a significant difference between groups (*p* < 0.05).

In the questionnaire survey, 115 farms responded (response rate: 99%). Of the 115 farms, 78% had changed their shipping strategy by voluntarily increasing the shipping weight, with the remaining 22% not changing their shipping strategy. The reasons given, in descending order of frequency, were insufficient space to extend the rearing period, the risk of decreased meat quality due to the longer rearing period, and shipping weights that were already set high before the CTSP revision. Table 1 shows the descriptive statistics of differences between 2022 and 2023 in the measurements of profitability and growth productivity. CWT was associated with CWT‐Change and CWT in 2022 (*p* < 0.05). The difference in CWT between 2022 and 2023 increased as CWT‐Change increased (Figure 2; *p* < 0.05). Farms with a CWT in 2022 of less than 74.3 kg exhibited a significantly higher difference in CWT between 2022 and 2023 than those with a CWT in 2022 of over 77.6 kg (*p* < 0.05; 1.3 ± 0.3 vs. 0.5 ± 0.2). No difference was found in the difference of feed cost per market pig between 2022 and 2023 among the CWT‐Change groups. However, farms that had increased the CWT by more than 2 kg in 2023 had a higher difference in the sales revenue per market pig than those with a decreased CWT in 2023 (*p* < 0.05). Consequently, farms that had increased CWT by more than 2 kg in 2023 had a higher difference in the margin over feed cost per market pig than those with a decreased CWT in 2023 (*p* < 0.05). Other measurements of profitability and growth productivity were not associated with CWT‐Change groups.

**TABLE 1:** Descriptive statistics of difference values between 2022 and 2023 in measurements of profitability and growth productivity on 116 farms and results of mixed‐effects model.

	Difference values between 2022 and 2023			Result of mixed‐effects model	
	Mean ± SD	25%	75%	CWT‐Change groups^a^	CWT in 2022 groups^b^
CWT (kg)	1.0 ± 1.4	0.1	1.9	*p* < 0.05	*p* < 0.05
Sales revenue per market pig (JPY)	3058 ± 1624	2044	3858	*p* < 0.05	0.44
Carcass price (JPY/kg)	33.1 ± 19.8	21.0	40.3	0.70	0.66
Feed cost per market pig (JPY)	1543 ± 1510	516	2592	0.72	*p* < 0.05
Feed price (JPY/kg)	3.0 ± 3.7	0.8	5.0	0.93	0.11
Margin over feed cost per market pig (JPY)	1514 ± 2096	58.7	2868.8	*p* < 0.05	0.52
Postweaning mortality (%)	0.2 ± 2.3	−1.1	1.0	0.20	0.73
Carcass‐based FCR	0.0 ± 0.3	−0.1	0.2	0.45	0.44
Age at slaughter (day)	4.0 ± 11.8	−4.8	11.7	0.88	0.52

^a^ The change in carcass weight per market pig (CWT) between 2022 and 2023 (CWT‐Change) was classified as farms that decreased CWT in 2023, farms that increased CWT by 0–1 kg in 2023, farms that increased CWT by 1–2 kg in 2023, and farms that increased CWT by over 2 kg in 2023.

^b^ The CWT in 2022 groups were classified based on the upper and lower 25th percentiles: less than 74.3 kg, 74.3–77.6 kg, and over 77.6 kg.

## 4 Discussion

The present study has revealed that the CTSP revision influenced the shipping strategy at many farms: 77% of farms voluntarily increased shipping weight and overall CWT significantly between 2022 and 2023 by approximately 1 kg, from 76.1 to 77.1 kg. Among the farms that increased the CTW in 2023, the weight gain values varied: one‐third exhibited an increase of less than 1 kg, another third an increase of 2 kg or more. However, 23% of farms had not increased the CWT. This percentage was consistent with the percentage of farms in the questionnaire survey that did not change their shipping strategy after the CTSP revision. Of the various reasons for not changing, the most frequently cited was having insufficient space to extend the rearing period. In recent years, with the introduction of highly prolific sows (Sasaki et al. 2020), many farms are experiencing insufficient housing space in their finishing barns. This type of farm may increase their CWT if they expand their rearing area. Another type of farm that answered that their shipping weights were already set high before the CTSP revision may trade directly between farmers and wholesalers without the JMGA (Terada et al. 2024).

The results of the present study have clarified that farms that increased CWT by over 2 kg increased their margin over feed cost per market pig compared with those that had not increased CWT. This is because farms that increased CWT by over 2 kg had higher sales revenue per market pig than those that had not increased CWT and had a similar feed cost per market pig to those that had not increased CWT. Similar studies have reported that a change in shipping strategy had a beneficial effect on farm businesses (Oliveira et al. 2015; Pourmoayed et al. 2016; Davoudkhani et al. 2020). Sales revenue per market pig consists of CWT and carcass price. In this study, no difference in the carcass price was found between the CWT‐Change groups, thus an increase in the margin over feed cost per market pig in farms that had increased CWT by more than 2 kg was considered to be an increase in CWT. However, contrary to our hypothesis, no difference was observed in the feed cost per market pig between the CWT‐Change groups. Regarding the age at slaughter, farms that increased CWT extended the age at slaughter in 2023 compared with 2022, but those that did not increase CWT also extended the age at slaughter. This might be because some farms that had not increased CWT had higher postweaning mortality and then an increased carcass‐based FCR and extended age at slaughter (Leen et al. 2018). This trend can be discerned from the box plot, although there was no statistically significant difference.

In this study, the differences in values between 2022 and 2023 in the measurements of profitability and growth productivity were used to minimize the effect of differences in the economic indicators between 2022 and 2023. Indeed, both the carcass price and feed price were significantly higher in 2023 compared with 2022. An increase in feed price would be caused by the Russian invasion of Ukraine and the depreciation in JPY (Ministry of Agriculture, Forestry and Fisheries of Japan 2025). These results thus indicate that attention must be paid to the changes in various indicators when conducting economic evaluations over several years (Mullan et al. 2011).

The present study had a limitation that should be noted when interpreting results. The carcass price was calculated by total sales revenue divided total CWT, and this study could not clarify the relationship with actual market prices and carcass grade. Further research is needed to determine this point.

In conclusion, the CTSP revision has probably led many farmers to intentionally increase their CWT, and farms that increased CWT by more than 2 kg increased their margin over feed cost compared with those that had not increased CWT. Our results also indicate that these findings could serve as a valuable benchmark for farms in determining their optimal CWT.

## Conflicts of Interest

The authors declare no conflicts of interest.
